# Traumatic brain injury: a comparison of diffusion and volumetric magnetic resonance imaging measures

**DOI:** 10.1093/braincomms/fcab006

**Published:** 2021-01-02

**Authors:** Niall J Bourke, Maria Yanez Lopez, Peter O Jenkins, Sara De Simoni, James H Cole, Pete Lally, Emma-Jane Mallas, Hui Zhang, David J Sharp

**Affiliations:** 1 Division of Brain Sciences, Computational, Cognitive and Clinical Neuroimaging Laboratory, Imperial College London, Hammersmith Hospital, London, UK; 2 UCL Department of Computer Science, Centre for Medical Image Computing, University College London, London WC1V 6LJ, UK

**Keywords:** diffuse axonal injury, diffusion tensor, neurite density, orientation dispersion, traumatic brain injury

## Abstract

Cognitive impairment after traumatic brain injury remains hard to predict. This is partly because axonal injury, which is of fundamental importance, is difficult to measure clinically. Advances in MRI allow axonal injury to be detected after traumatic brain injury, but the most sensitive approach is unclear. Here, we compare the performance of diffusion tensor imaging, neurite orientation dispersion and density-imaging and volumetric measures of brain atrophy in the identification of white-matter abnormalities after traumatic brain injury. Thirty patients with moderate–severe traumatic brain injury in the chronic phase and 20 age-matched controls had T1-weighted and diffusion MRI. Neuropsychological tests of processing speed, executive functioning and memory were used to detect cognitive impairment. Extensive abnormalities in neurite density index and orientation dispersion index were observed, with distinct spatial patterns. Fractional anisotropy and mean diffusivity also indicated widespread abnormalities of white-matter structure. Neurite density index was significantly correlated with processing speed. Slower processing speed was also related to higher mean diffusivity in the corticospinal tracts. Lower white-matter volumes were seen after brain injury with greater effect sizes compared to diffusion metrics; however, volume was not sensitive to changes in cognitive performance. Volume was the most sensitive at detecting change between groups but was not specific for determining relationships with cognition. Abnormalities in fractional anisotropy and mean diffusivity were the most sensitive diffusion measures; however, neurite density index and orientation dispersion index may be more spatially specific. Lower neurite density index may be a useful metric for examining slower processing speed.

## Teaser

There are various methods to assess white-matter damage after traumatic brain injury, including neurite orientation and dispersion imaging. Bourke *et al.* found that volumetric differences were the most sensitive to group differences between patients and controls. However, diffusion measures provide greater specificity in relation to cognitive performance after moderate–severe brain injury.

## Graphical Abstract

**Graphical Abstract:**
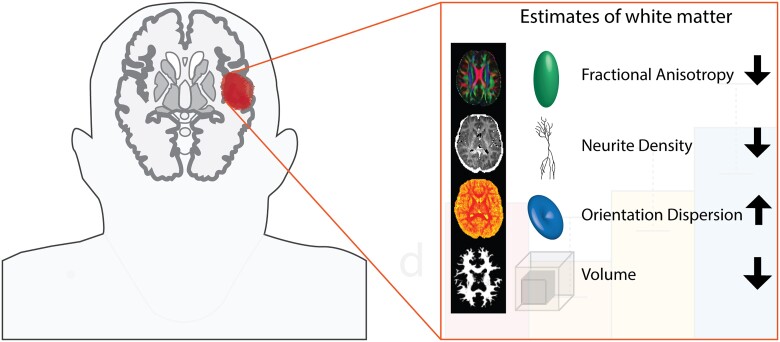

## Introduction

Outcomes after traumatic brain injury (TBI) are often poor and remain hard to predict (Maas *et al.*, 2017). A major reason for this is the difficulty in determining the degree of underlying brain injury. In particular, diffuse axonal injury (DAI) has a key role in the pathophysiology of TBI but it is difficult to measure clinically. Due to sheering forces experienced at the time of injury, widespread damage to axonal membranes can occur (Sharp *et al.*, 2014; Ghajari *et al.*, 2017). Conventional diagnostic imaging approaches such as visual inspection of CT and standard MRI often underestimate the severity of DAI. Susceptibility weighted imaging is sensitive to diffuse vascular injuries, but diffusion imaging provides distinct information about the microstructure of the white matter (WM), which is disrupted by DAI and often appears normal on standard imaging (Kinnunen *et al.*, 2011; Jolly *et al.*, 2021). Major advances have been made over the last decade in the use of magnetic resonance imaging (MRI) to quantify post-traumatic axonal injury; however, the most sensitive approach is unclear.

Diffusion-MRI has been widely used to assess WM structure after TBI (Mac Donald *et al.*, 2007) (Kinnunen *et al.*, 2011). The diffusion properties of water molecules within WM tracts provide information about their structure. Diffusion tensor imaging (DTI) has been used most commonly; fitting a tensor model at each voxel allows a range of metrics to be calculated such as fractional anisotropy (FA), which is frequently used as a marker of WM disruption (Mac Donald *et al.*, 2007). However, measures derived from a single tensor are unlikely to adequately describe the complex patterns of underlying WM pathology. Multi-shell diffusion-MRI promises a more detailed description of WM pathology associated with DAI by allowing more flexible analysis. For example, Neurite Orientation Dispersion and Density Imaging (NODDI) models three compartments that are characterized by distinct diffusion properties: intra-neurite (axons and dendrites) characterized by restricted diffusion; extra-neurite (cell bodies and glia) characterized by hindered diffusion and cerebrospinal fluid (CSF) characterized by free diffusion (Zhang *et al.*, 2012). These are labelled as neurite density index (NDI), orientation dispersion index (ODI) and isotropic volume fraction (ISOVF), respectively. Volume measures were also frequently used when investigating clinical populations. Progressive WM atrophy is seen in the chronic phase of injury after TBI, which is sensitively quantified with MRI, providing a measure of neurodegeneration (Cole *et al.*, 2018). In the chronic phase after TBI, volumetric measures of WM structure can be a sensitive, albeit non-specific indicator of previous DAI.

Here, for the first time, we compared the performance of DTI, NODDI and volumetric measures of WM structure in the identification of WM abnormalities after moderate–severe TBI. We tested whether lower NDI and higher ODI are seen in patients with TBI compared to healthy controls. We then compared the sensitivity of NODDI metrics to FA, a more standard measure of diffusion MRI, and to volumetric measures of WM structure. We then assessed whether the spatial patterns of alterations are distinct from each other. Finally, we also investigated how these NODDI metrics relate to cognitive function and compared their sensitivity for detecting cognitive impairment with FA.

## Methods

### Study participants

Thirty-one patients with moderate–severe TBI (26 male, mean age ± SD = 38.5 ± 10.1) and 20 age-matched controls (17 male, mean age ± SD = 37.85 ± 10.74) were recruited (Supplementary Table 2). One patient was excluded due to motion artefacts leaving 30 patients for further analysis. Mechanism of injury varied across participants: Road Traffic Accident (16), Assault (7), Falls (5) and Other (2) (Supplementary Table 2). All patients recruited in the post-acute/chronic phase (median time since injury 34 months, range 6–360 months) were scanned at the Clinical Imaging Facility, Imperial College London. Patients were recruited through specialist TBI outpatient clinics in London or referred from their local brain injury service based on on-going functional and/or cognitive impairment. Severity of injury was based on the Mayo classification system (Malec *et al.*, 2007). This considers the duration of loss of consciousness, post-traumatic amnesia, lowest recorded Glasgow coma scale and neuroimaging. Pre-morbid psychiatric and neurological illnesses were exclusion criteria, along with contraindication to MRI. The study was approved by the West London and GTAC NRES Committee (14/LO/0067). All participants provided informed consent written consent and were screened for capacity by a neurologist. A consultant neuroradiologist reviewed all structural MRI scans.

### Neuropsychological assessment

Participants completed a standard neuropsychological battery to investigate cognitive domains commonly associated with dysfunction after traumatic brain injury. Specific measures were selected based on the previous study to investigate potential relationships to WM brain structure (Kinnunen *et al.*, 2011). These measures include: (i) processing speed measured by a computerized choice reaction time (CRT) task; (ii) alternating switch cost index from the trail-making task, alternating between letters and numbers—numbers only; (iii) Delis–Kaplan Executive Function System Colour-Word Interference Test (Stroop; Delis *et al.*, 2001) and (iv) Wechsler Memory Scale (WMS-III) logical memory, delayed recall (Wechsler, 1945). Intellectual ability diverges from impairment of specific cognitive domains. Previous findings suggest that estimates of pre-morbid ability can be ascertained through the measures of matrix reasoning, as this is often spared after TBI (Donders *et al.*, 2001).

### Image acquisition

MRI was performed on a Siemens Verio 3.0 Tesla scanner using a 32-channel head coil. Each patient had standard high-resolution structural imaging, acquired with the following parameters: T1 MPRAGE (TE = 2.98 s, TR = 2.3 s, 1 mm isotropic voxel, 256 × 256 mm field of view, FA = 9, GRAPPA = 2, 5 min scanning time), T2 FLAIR (TE = 395 ms, TR = 5 s, 1 mm isotropic voxel, 250 × 250 mm field of view, GRAPPA = 2, 6 min scanning time). For diffusion-MRI, the NODDI multi-shell protocol included one shell with 30 gradient directions and *b* = 700 s/mm^2^ and another with 60 directions and *b* = 2000 s/mm^2^. The protocol also contained nine images without diffusion weighting (*b* = 0 s/mm^2^) and a single reversed-phase encoding image without diffusion weighting. The EPI multiband readout (multiband factor = 3, TE = 105.2 ms, TR = 5 s) used a matrix size of 128 × 128 over a field of view of 256 × 256 mm^2^ and slice thickness of 2 mm, resulting in isotropic voxels of 2 mm^3^. A total of 66 contiguous slices were acquired for whole-brain coverage. The total scanning time for the NODDI protocol was 10 min. An additional single-shell acquisition (64 directions, *b* = 1000 s/mm^2^) was acquired for comparison.

### Image processing and analysis

**Figure 1:**
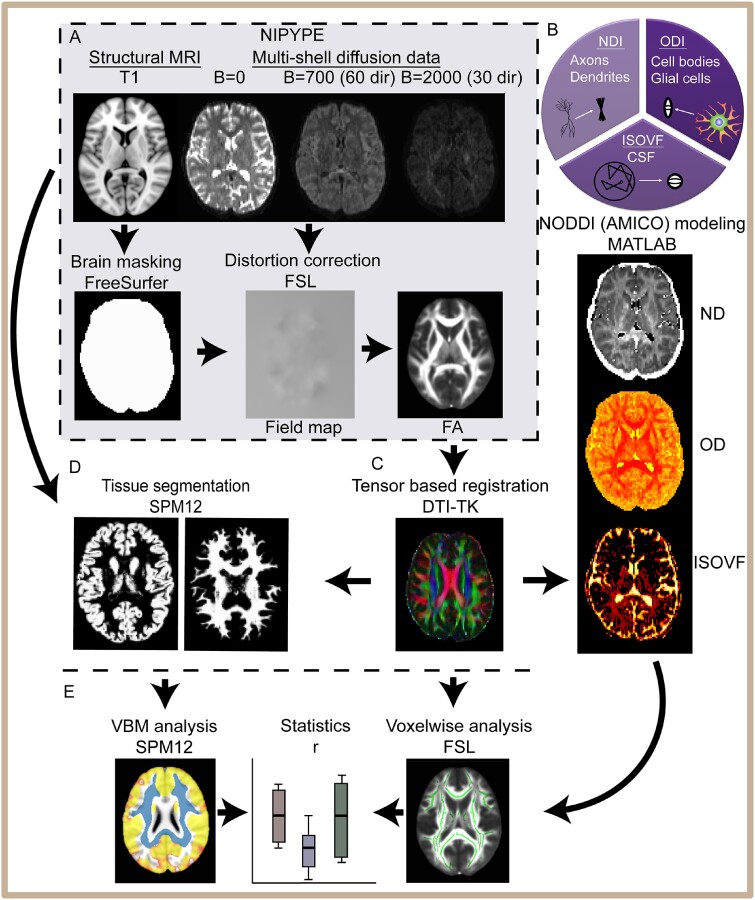
**Neuroimaging analysis pipeline.** (**A**) An automated NIPYPE pipeline-extracted structural and diffusion data from XNAT and ran pre-processing including FreeSurfer segmentation for structural T1 and eddy, top-up distortion correction for diffusion-weighted images. Standard DTI metrics are generated with FSL within the NIPYPE framework. (**B**) NODDI modelling of diffusion data used MATLAB. NODDI produces the measures of three compartments: intra-neurite (axons and dendrites) characterized by restricted diffusion; extra-neurite (cell bodies and glia) characterized by hindered diffusion and CSF characterized by free diffusion. (**C**) Registration is performed with DTI-TK. (**D**) Voxel-based morphometry analysis with SPM12, segmenting T1-weighted images into grey and WM probability maps. These are normalized and smoothed (8 mm). (**E**) Finally, voxel-wise statistical analysis was carried out using FSL TBSS. Summary measures were exported into R for statistical analysis.

Image processing and analysis was performed using a variety of packages, partially implemented through a NIPYPE (Neuroimaging in python, NIPY) pipeline to automate the process and ensure reproducibility. A high-level overview of the analysis pipeline is shown in Fig. 1. NIPYPE is an open-source, community-developed Python project that provides a uniform interface to existing neuroimaging software and facilitates interaction between these packages within a single workflow. The pre-processing NIPYPE pipeline included segmentation of structural T1-weighted data and the removal of non-brain voxels using Freesurfer; correction of susceptibility induced distortions, eddy current distortions and rigid-body head motion in diffusion-MRI data, using the tools Topup and Eddy from FMRIB Software Library image processing toolbox FSL (Smith *et al.*, 2004; Woolrich *et al.*, 2009). dMRI data were then analysed to extract standard DTI metrics (FA, MD) using FSL dtifit (Behrens *et al.*, 2003) from both the multi-shell and the single-shell data.

NODDI modelling and DTI analysis were run in parallel on the multi-shell DWI. Tensor-based registration using DTI-TK (Zhang *et al.*, 2006) was performed on the processed dMRI data from NIPYPE generating DTI metrics (FA, MD). Tract-based spatial statistics (TBSS) was then performed, as per our previous work (Bonnelle *et al.*, 2012). NODDI modelling was performed using the Accelerated Microstructure Imaging via Convex Optimization framework (Daducci *et al.*, 2015), which accelerates the fit up to four orders of magnitude by re-formulating the model as a linear system, preserving accuracy and precision in the results. The output of this modelling produces NDI, ODI and ISOVF images which were then moved into the standard space for voxel-wise analysis.

Brain tissue volumes, WM, grey matter and intra-cranial volume were computed for each individual using a standard morphometry pipeline on T1-weighted images with (SPM12, University College London, www.fil.ion.ucl.ac.uk/spm (27 February 2021, date last accessed)). These criteria were described previously in more detail in the study of Cole *et al.* (2018).

### Statistical analysis

Voxel-wise analysis of the NODDI metrics (NDI, ODI and ISOVF) and multi-shell-derived DTI measures (FA, MD) were performed using TBSS in the FMRIB Software Library (Smith *et al.*, 2004, 2006). The mean FA image was constrained to produce a ‘skeleton’, showing WM tracts centres, and therefore reducing partial-volume confounds. The FA skeleton was subsequently set to a threshold of ≥0.2 to suppress regions of extremely low mean FA and to remove areas with substantial inter-individual variability. This was saved as a binarized mask for subsequent statistical analysis. Equivalent steps for processing non-FA images were then performed to derive the MD and NODDI images. Independent sample *t*-tests were run to investigate differences in diffusion metrics between patients and controls, predicting that TBI would produce reduced FA, NDI, volume and increased MD and ODI. This was done with non-parametric permutation testing (*n* = 10 000) in FSL Randomise (Winkler *et al.*, 2014). A threshold of *P* < 0.05 was then applied on the FWE-corrected results. Analysis of the single-shell DWI acquisition followed the same analysis pipeline (Supplementary Fig. 1). The same permutation testing was applied to the measures of WM volume derived from SPM12. Neuropsychological test results were included in separate voxelwise analysis of each metric to assess relationship with WM structure. Age and gender were included as nuisance regressors in all analyses with the addition of intra-cranial volume for VBM analysis. Analysis of behavioural tests and summary dMRI measures was conducted using the R statistical environment (R Core Team, 2018; http://www.R-project.org/ (27 February 2021, date last accessed)).

### Lesion segmentation

Semi-automatic segmentation, using IMSEG v1.8, was conducted to delineate brain areas with focal lesions. Segmentation is based on an algorithm for geodesic image segmentation as described in the study of Criminisi *et al.* (2008). T1-weighted and FLAIR images were imported into the software and co-registered. Lesion maps were drawn as overlays on the T1-weighted images, using FLAIR to improve contrast for accuracy. To generate the lesion probability distribution, binary lesion masks were transformed to MNI standard space using Advanced Normalisation Tools (Avants *et al.*, 2011) followed by concatenation of masks to display the regions of increasing lesion burden using FSL lesion tools.

## Data availability

Data are available on request from the authors.

### Results

**Figure 2:**
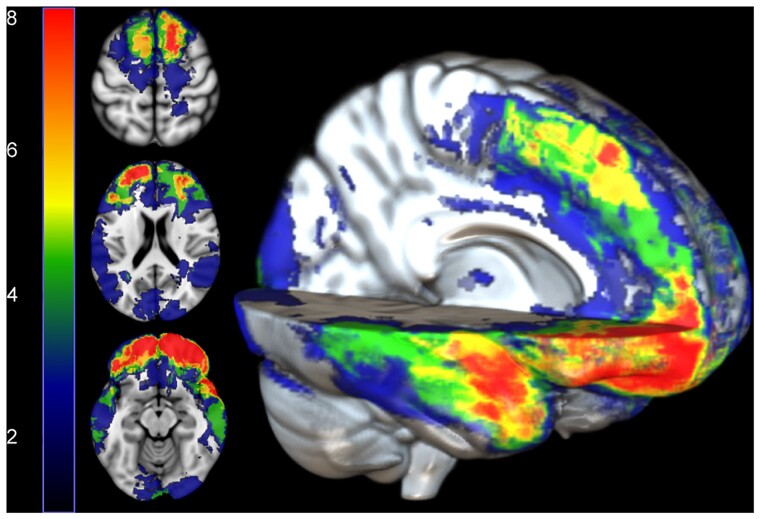
**Lesion distribution map.** Regions indicating greatest lesion burden in red. Numbers on the colour bar represent the number of TBI patients with a lesion at that voxel.

Focal brain lesions were found in 83% of the patients (Fig. 2). The highest areas of overlap were seen in the orbital frontal cortex, superior parts of the medial prefrontal cortex and the temporal poles. A comparison of moderate–severe lesion (*n* = 20) and non-lesion TBI (*n* = 10) patients showed no difference between the measures of reaction time (*W* = 115, *P*_unc._ = 0.33) and delayed memory recall (*W* = 81, *P*_unc_ = 0.41) or executive function measured by DKEFS Stroop (*W* = 123, *P*_unc._ = 0.31). Cognitive performance is discussed in more detail in relation to diffusion measures below. A summary of neuropsychology performance is provided in Supplementary Table 1.

### Abnormalities in NODDI metrics after TBI

**Figure 3:**
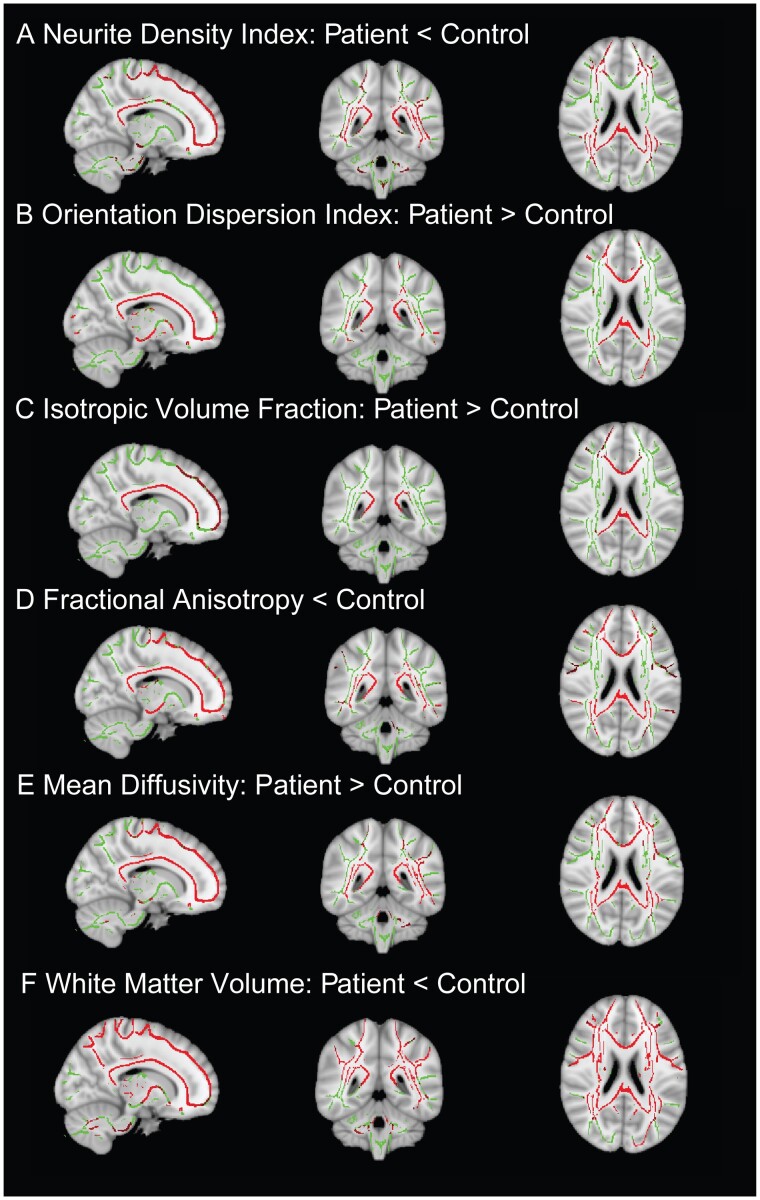
**Widespread WM disruption in imaging measures after TBI.** Whole-brain TBSS contrasts between patients with TBI and controls. Red: Voxels with significant differences between patients and controls. Contrasts are overlaid on the mean FA skeleton (green) and are adjusted for age, gender and intracranial volume (TFCE: *P* < 0.05, corrected for multiple comparisons).

Widespread abnormalities were observed in the diffusion measures produced by NODDI modelling. Reductions in NDI were seen in a large number of WM tracts (Fig. 3A), including the genu and splenium of the corpus callosum and the inferior longitudinal fasciculus bilaterally. ODI showed the reverse pattern, with higher values in TBI patients compared with controls (Fig. 3B). Abnormally high ODI was seen bilaterally in the cortical spinal tracts and body of the corpus callosum, with further reductions seen in the splenium and genu of the corpus callosum and bilateral inferior longitudinal fasciculus. ISOVF showed higher values in patients with TBI in all sections of the corpus callosum, as well as anterior parts of the fronto-occipital tracts. There were no WM tracts, showing higher NDI or lower ODI or ISOVF in the patient group.

### Diffusion tensor imaging abnormalities

DTI results presented are from the same multi-shell acquisition as the modelled NODDI data. As expected, lower FA was seen in patients after TBI compared to age-matched controls in widespread WM regions (Fig. 3D). These regions included the inferior longitudinal fasciculi, inferior frontotemporal occipital fasciculi, corticospinal tracts and all parts of the corpus callosum. MD was higher in patients, showing a similar spatial pattern to that observed with FA (Fig. 3E). There were no WM tracts, showing higher FA or lower MD in the patient group compared to the control group.

### Volumetric analysis

**Figure 4:**
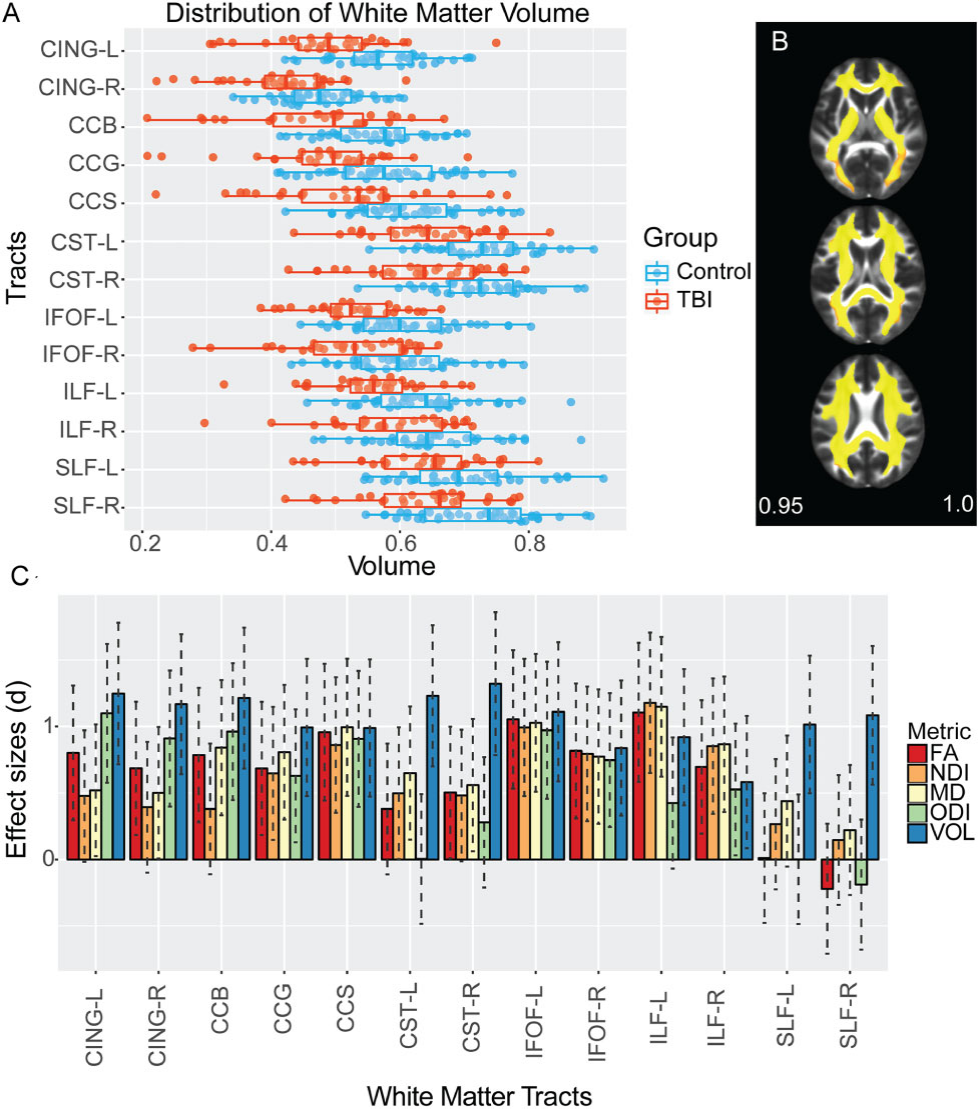
**Effect size across WM measures.** (**A**) Distribution of WM volume across multiple tracts. Volume calculated using JHU atlas for parcellation of tracts. (**B**) Voxels showing significantly (*P* < 0.05) low WM (yellow–red) volume in patients compared to controls. (**C**) Plot of effect size with confidence intervals between patients and controls across metrics for different tracts. FA (red), NDI (orange), MD (yellow), ODI (green) and volume (VOL, blue). CING, cingulate; CCG, corpus callosum genu; CCS, corpus callosum splenium; CST, cortico-spinal tract; IFOF, inferior fronto-occipital fasciculus; ILF, inferior longitudinal fasciculus; SLF, superior longitudinal fasciculus.

There was markedly reduced total WM volume in the TBI group [mean = 0.435 (0.06)] compared with controls [mean = 0.497 (0.06)], *t* = 4.115, *P* < 0.001. Voxelwise analysis showed evidence of lower WM volume (atrophy) across most of the WM (Figs. 3 and 4B). When analysing WM tracts individually (Fig. 4A), lower volumes were apparent in all sampled tracts including the corpus callosum and bilateral superior and inferior longitudinal fasciculi for patients compared with controls.

### Effect sizes for white-matter abnormality detection

We next compared the effect sizes for the ability of diffusion and volumetric measures to discriminate between patients and controls, using Cohens *d*. Medium and large effect sizes were observed with varying patterns across NDI, ODI, FA, MD and volume (Fig. 4C). The splenium of the corpus callosum had similar large effect sizes (*d* > 0.8) across all diffusion metrics and volume, as did the inferior fronto-occipital fasciculus bilaterally. NDI did not show a significant effect in the body of the corpus callosum, whereas it was detected by other diffusion metrics and volume. The largest effect sizes were seen for volumetric measures. All tracts showed significant reductions in volume, including tracts that showed no change in any of the diffusion measures studied. This was particularly apparent for the superior longitudinal fasciculus where large effect sizes for volumetric reduction were accompanied by no significant differences in any diffusion-MRI measures. Large effects for volume reduction were seen in the corticospinal tract, which was accompanied only with increased MD. Comparative analyses of DTI metrics with diffusion data acquired with a single shell for these participants were run. This showed comparable effect size patterns across tracts for single-shell FA and MD to the multi-shell results.

### Relationship between neuroimaging measures

**Figure 5:**
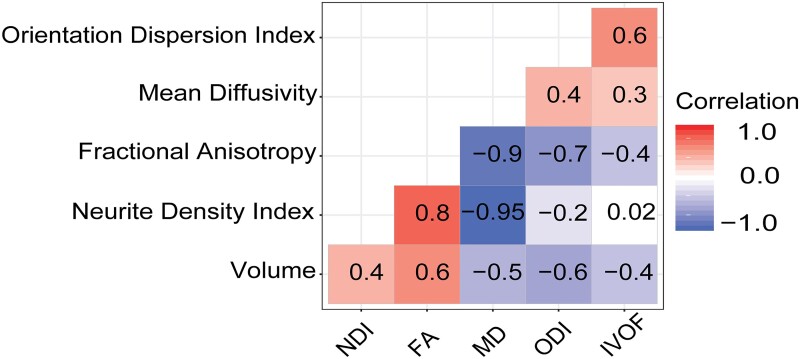
**Correlation matrix of diffusion metrics and WM volume.** Red, positive correlation; blue, negative correlation and white, not significant. The *y*-axis is ordered based on hierarchal clustering with increasing correlation coefficients.

Strong correlations were generally present between diffusion and volumetric measures, calculated from the whole-WM skeleton (Fig. 5). There was a strong positive correlation between NDI and FA (*r* = 0.83, P < 0.001) and a negative correlation between ODI and FA (*r* = −0.72, *P* < 0.001) and ISOVF and FA (*r* = −0.39, *P* < 0.001). These relationships suggest that the signal from FA consists of both the elements of neurite density and the orientation of the fibres. MD was also negatively correlated with both FA (*r* = −0.88, *P* < 0.001) and NDI (*r* = −0.95, *P* < 0.001). There were also significant positive correlations between WM volume (WM_vol_) and NDI (*r* = 0.42, *P* < 0.001) and WM_vol_ and FA (*r* = 0.63, *P* < 0.001), with negative correlations between WM_vol_ and ODI (*r* = −0.65, *P* < 0.001), WM_vol_ and ISOVF (*r* = −0.39, *P* < 0.001) and WM_vol_ and MD (*r* = −0.50, *P* < 0.001).

### Relationships between neuroimaging measures and cognitive function

**Figure 6:**
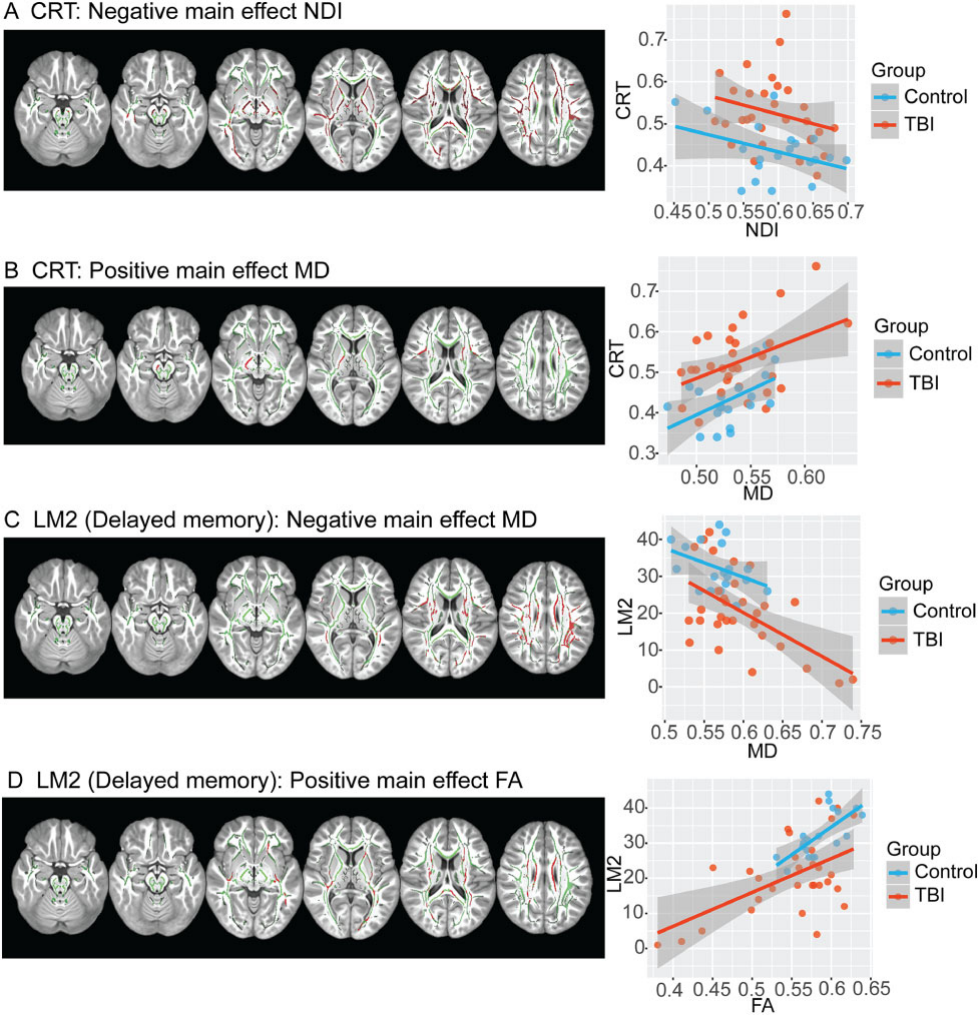
**Correlation of neuropsychological assessment with MRI measures of WM.** (**A**) Voxels with a negative correlation between NDI and CRT (red); (**B**) Voxels with a positive correlation between mean diffusivity and CRT; (**C**) Voxels with a negative correlation with delayed recall logical memory; (**D**) Voxels with a positive correlation and delayed recall on logical memory. Contrasts are overlaid on the mean FA skeleton (green) and are adjusted for age, gender and intracranial volume (TFCE: *P* < 0.05, corrected for multiple comparisons). Scatterplots illustrate the mean intensity values of significant voxels against cognitive performance for each of the tests.

There were significant relationships between diffusion measures and neuropsychological performance. NDI was significantly correlated with processing speed, as indexed by CRT response, in extensive WM regions. Increasing reaction times on the CRT (worse performance) were associated with reductions in NDI within large parts of the WM including the corpus callosum, cingulum and inferior longitudinal fasciculus. This relationship was not seen for ODI or ISOVF. There were no significant relationships between other neuropsychological measures and either ODI, NDI or ISOVF. The performance of CRT was also correlated positively with MD, primarily within the corticospinal tracts (Fig. 6). FA and MD were correlated with delayed recall for associative memory (Wechsler, 1945). Lower FA values were indicative of poor memory recall across both patient and control groups, whereas patients were further along with the distribution with lower scores. Similarly, an inverse relationship for MD was present, with lower scores of memory recall being associated with higher MD. Significantly lower FA was seen within the body of the corpus callosum and cingulate. Higher MD was also seen in these tracts. Significant relationships were not observed between diffusion measures and any other neuropsychological results. There were also no significant relationships seen between volumetric measures and any neuropsychological results.

## Discussion

This study applies the advanced diffusion NODDI model to the investigation of WM microstructural changes in moderate–severe TBI for the first time. Advances in magnetic resonance imaging provide new ways to investigate axonal injury after TBI. We applied this NODDI model in patients with persistent neurological problems after TBI alongside two widely used measures of WM structure, diffusion tensor imaging and volumetric measures of brain atrophy, with a neurite orientation dispersion and density-imaging model. Widespread abnormalities in all three were observed, particularly in midline structures such as the corpus callosum that are particularly affected by DAI (Ghajari *et al.*, 2017). Reductions in brain volume were most sensitive in identifying abnormality after TBI, as measured by effect sizes differentiating age-matched controls from patients with TBI. This non-specific measure was abnormal across all of the large WM tracts investigated, showing consistently large effect sizes across all the tracts. Medium-to-large effect sizes were also seen for the diffusion measures in most of the WM tracts studied.

Discrepancies were observed between volumetric and diffusion measures in some tracts. Most notable was the absence of any diffusion abnormalities in the superior longitudinal fasciculi, but the presence of abnormally low volumes in this tract bilaterally. This discrepancy suggests that diffusion measures show various sensitivities to underlying pathologies, in this case, demonstrated as reduced tract volume. One explanation for a spatial variation in sensitivity may be a physical limitation in diffusion-MRI acquisition. Diffusion metrics are known to be spatially heterogeneous across brain regions, with the highest signal-to-noise in medial parts of the brain (Jones and Cercignani, 2010). This would be expected to impact on the sensitivity of diffusion measures to pathology, which is often assumed to be uniform. Hence, diffusion metrics from lateral parts of the brain may show reduced sensitivity to pathology. T1 structural scans are acquired at a higher resolution (1 mm^3^) to diffusion-MRI (2 mm^3^). With this higher resolution, there is potentially more power to pick up group differences.

We also investigated the relationship between imaging measures and cognitive performance. Individual differences in volumetric measures did not correlate with variation in cognitive performance across the patient group. In contrast, individual differences in the amount of WM damage quantified by diffusion measures correlated with cognitive performance. FA and MD have been shown to relate to cognitive performance after TBI (Bonnelle *et al.*, 2012; Hellyer *et al.*, 2013) and similar relationships were again observed. A clear relationship was observed between neurite density and information processing speed, suggesting that the loss of neuronal elements may be particularly important for the processing speed impairments that are characteristically seen after TBI. No differences are seen in cognitive performance between patients classified as moderate–severe with and without focal lesion. While beyond the scope of this study, a detailed investigation of the interaction of focal lesions and WM damage after TBI would be informative.

DTI has been widely used to study axonal injury after TBI. Though sensitive to WM abnormalities, FA lacks specificity, reflecting a combination of axon density, axon distribution, gliosis, oedema and degree of myelination. This limits clear interpretation about pathological mechanisms underlying axonal injury and associated cognitive deficits after TBI. Histological analysis of animal models suggests that NODDI metrics NDI and ODI may provide a better representation of the biological microstructure than FA (Jespersen *et al.*, 2010; Levine and Schweitzer, 2014; Sepehrband *et al.*, 2015; Grussu *et al.*, 2017; Sato *et al.*, 2017). ODI has been validated as a suitable index of tissue microstructure, with higher values in the areas of crossing fibres compared to parallel fibres in different areas of the mouse brain (Sato *et al.*, 2017). In this study, there are widespread abnormalities in ODI and NDI in a moderate–severe TBI group. Distinct spatial abnormalities are seen across different tracts and diffusion metrics. Despite both DAI and processing speed being commonly affected after TBI, there has been a surprising lack of relationship between these measures in the previous study (Kinnunen *et al.*, 2011). Here, we found a widespread relationship with lower neurite density and slower processing speed. However, no relationship was present for ODI. These results suggest that NODDI can be used to clarify the location and extent of WM damage, in such a way that it is relevant to improving our understanding of post-traumatic cognitive impairment. Recent work provides evidence that reductions in neurite density are related to a combination of reduced numbers of neuronal elements and demyelination of damaged axons, whereas increased ODI may be associated with axonal disorganization (Kamiya *et al.*, 2020). DAI can produce progressive neurodegeneration and chronic demyelination (Armstrong *et al.*, 2016; Cole *et al.*, 2018), potentially explaining the reduction in neurite density. The loss of axons within a WM tract and the demyelination of damaged axons within that tract would be expected to lead to a slowing of conduction velocity through the tract as a whole. This provides a direct mechanistic explanation for slow information processing for cognitive functions supported by brain networks that include the damaged tract. DAI can also produce axonal disorganization, that is sensitively identified by ODI change (Donat *et al.*, 2021). However, it is plausible that this change in tract structure might not directly change conduction velocities and hence impact less of information processing speed.

Distinct patterns of NDI and ODI changes were observed in different WM tracts, disassociating the contribution of signal for FA changes. ODI was significantly higher in the body of the corpus callosum body, whereas there were no significant NDI differences. However, changes in both NDI and ODI could be seen in the genu and splenium of the corpus callosum. An increase in ODI in the corpus callosum, an area which is protected from the effects of a direct impact, might be associated with the high strain rates caused by the biomechanics of TBI (Viano *et al.*, 2005). Evidence that the corpus callosum undergoes greater shear forces after trauma has been previously shown in a computer model of fall-induced TBI (Ghajari *et al.*, 2017).

Although there is a high degree of correlation between NODDI and DTI metrics, diverse effects can be seen in spatially distinct tracts. Globally, NDI has a strong negative relationship with MD (variance, 95%) and weak relationship to ODI. On average, NDI may not be much more informative that MD, however, ODI potentially offers novel information about WM structure than can be obtained from DTI metrics. The relationship between WM volume and NDI appeared surprisingly low (*r* = 0.4), whereas compared to ODI with volume there was a moderate negative relationship (*r* = −0.6). Reduction in WM volume is commonly seen after TBI, but it remains unclear what mechanism is driving this atrophy.

CSF contamination can produce errors in diffusion metrics, causing FA to be underestimated and reducing tissue characterization accuracy by as much 60% (Salminen *et al.*, 2016). One advantage of NODDI over DTI is that CSF is accounted for, by being modelled as a separate compartment of the signal. Hence, partial volume effects from CSF may less influence NODDI metrics, which might be particularly beneficial in areas of brain atrophy. An increase in CSF contribution to the signal would result in an increase in the ISOVF, leaving the other two metrics (NDI and ODI) unaffected (Colgan *et al.*, 2016). By combining NODDI with the ‘skeletonization’ process in TBSS, the impact of partial volume effects is reduced still further, particularly important in conditions associated with atrophy such as moderate–severe TBI (Cole *et al.*, 2018).

A main consideration during the development of NODDI was its potential clinical feasibility (Zhang *et al.*, 2012). The multi-shell acquisition is increasingly becoming more accessible and it comes with little extra time cost compared with traditional single-shell acquisitions. Furthermore, optimized NODDI processing means that a single brain can be analysed in under 10 min (Daducci *et al.*, 2015). As more information can be gathered from the diffusion properties of different brain tissues via a multi-shell acquisition, it promises to be increasingly useful in clinical settings.

The main limitation of the NODDI model is the assumption of a single intrinsic diffusivity, which is the microscopic diffusion coefficient parallel to the neurites, across the whole brain, with a fixed value of 1.7 μm^2^/ms for *in vivo* human studies (Zhang *et al.*, 2012; Daducci *et al.*, 2015). However, recent work has shown that this parameter to vary over different regions/age ranges in the healthy brain (Kaden *et al.*, 2016). This study did not account for CSF partial volume and may not be comparable. In contrast, other current study suggests that in adults this fixed value of 1.7 μm^2^/ms is optimal in WM but it is lower in grey matter and would require optimization (Guerrero *et al.*, 2019). Pathology-induced variations could also introduce further variations in the case of TBI. Another limitation of the model is that it does not account for multiple crossing fibres. This has previously been highlighted as an issue for DTI metrics such as FA. Here, it is a potential confound for ODI but NDI should remain unbiased. Here, we were unable to make a direct comparison with underlying histological measures. Hence, caution is needed in the interpretation of NDI and ODI as the exact relationship with underlying biology remains uncertain. Although some histopathological evidence has been provided to validate the assumptions of advanced diffusion metrics using animal models, to date, this has been limited; there is scope for further clinical and pre-clinical work. We did not perform multiple comparison correction across the different imaging measures, as our analysis explored whether these metrics provided independent information. ‘There was a small difference in the gender composition of the patient and control groups’. However, this is unlikely to have influenced our results, as gender makes only a small contribution to the variance associated with the effects of TBI on brain atrophy (Cole *et al.*, 2018) and we corrected for intra-cranial volume, which also controls for some gender-associated variability (Voevodskaya *et al.*, 2014).

## Conclusion

We observed abnormalities in NODDI metrics after TBI that can be decomposed into partially overlapping changes. Neurite density shows a strong relationship with processing speed, a useful measure of cognitive function after TBI. However, ODI appeared to provide a more distinct measure compared to other diffusion metrics, which may potentially provide additional estimates of the underlying neuropathology seen after TBI. Overall volume changes demonstrated to be the most sensitive marker and could be used as a target for interventions. There is value in using advanced techniques such as multi-shell diffusion-MRI. Through these advanced methods, improvements in disentangling the biological mechanisms underlying the DAI signal measured with diffusion-MRI can be made. This will be important for uncovering the neurobiological and cognitive changes that are associated with TBI as well as developing treatments and predicting outcomes.

## Supplementary material

Supplementary material is available at *Brain Communications* online.

## Funding

This article presents independent research funded by a National Institute of Health Research Professorship (NIHR-RP-011–048) awarded to D.J.S. and supported by the National Institute of Health Research Clinical Research Facility and Biomedical Research Centre at Imperial College Healthcare National Health Service Trust & National Institute of Health Research Clinical Research Facility. The views expressed are those of the author(s) and not necessarily those of the National Health Service, the National Institute of Health Research or the Department of Health. N.J.B. is funded by Imperial Presidents PhD scholarship.

## Competing interests

The authors report no competing interests.

## Supplementary Material

fcab006_Supplementary_Data

## Abbreviations

CRT =: choice reaction time
CSF =: cerebral spinal fluid
DAI =: diffuse axonal injury
DTI =: diffusion tensor imaging
FA =: fractional anisotropy
MD =: mean diffusivity
MRI =: magnetic resonance imaging
NDI =: neurite density index
NIPYPE =: neuroimaging in python
NODDI =: neurite orientation dispersion and density imaging
ODI =: orientation dispersion index
TBI =: traumatic brain injury
TBSS =: tract-based spatial statistics
WM =: white matter
