# Supplementary material for: Traumatic brain injury: a comparison of diffusion and volumetric magnetic resonance imaging measures
Source: Brain Commun. 2021 Jan 2;3(2):fcab006. doi: 10.1093/braincomms/fcab006 (PMC8105496; doi:10.1093/braincomms/fcab006)
Supplement: fcab006_Supplementary_Data [file fcab006_supplementary_data.pdf]

Supplementary material for Traumatic brain injury: a comparison of diffusion and volumetric magnetic resonance imaging measures

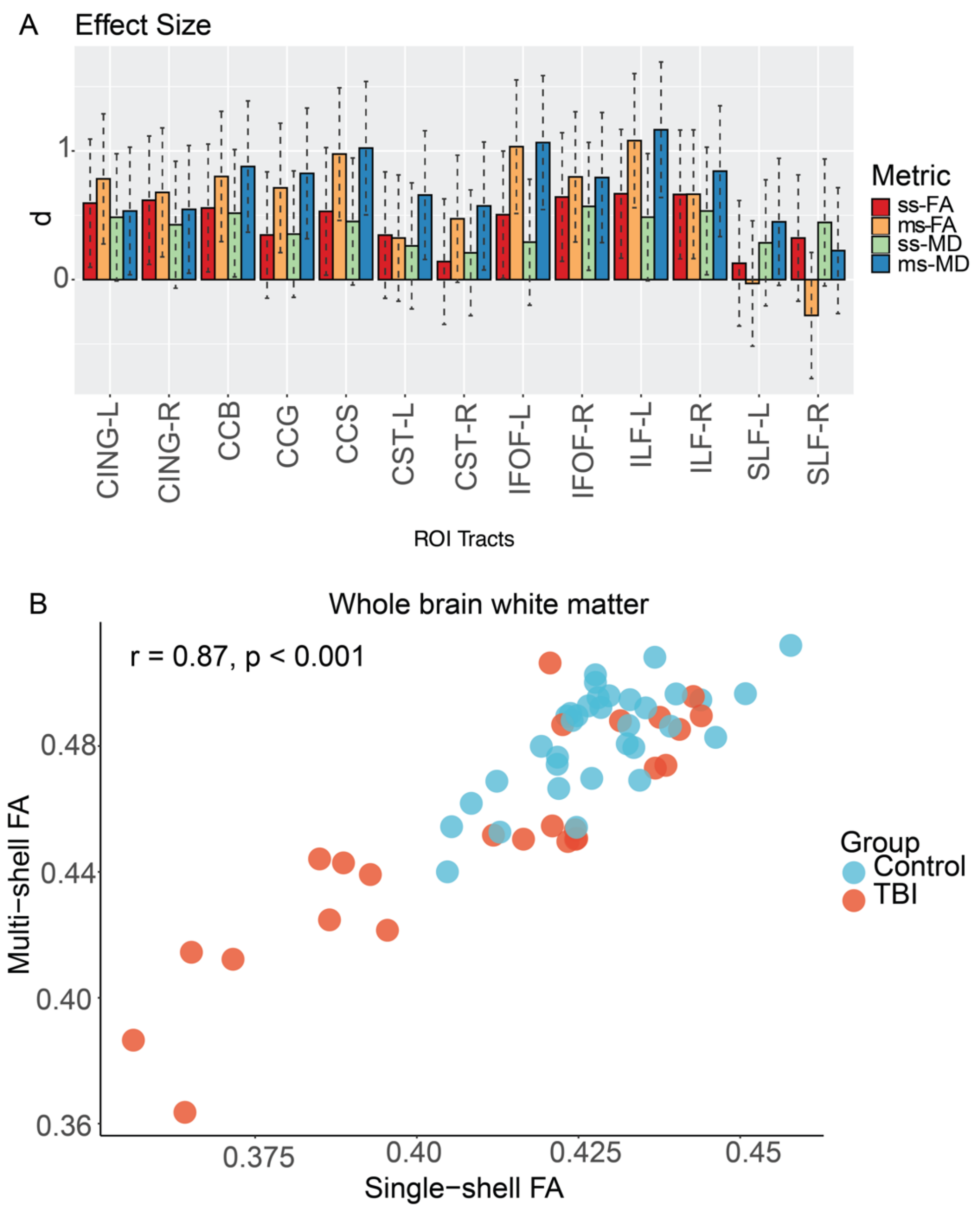

Supplementary Figure 1: Comparison of single & multi-shell metrics. A) Effect sizes for single (ss) & multi-shell (ms) FA and MD. B) Relationship of whole brain white matter skeleton for single & multi shell FA.

Supplementary Table 1. Neuropsychological measures in healthy controls and traumatic brain injury patients. Independent sample t-tests were conducted with the Wilcoxon Rank Sum Test with FDR multiple comparison correction between all pairwise means. WASI = Wechsler Abbreviated Scale for Intelligence; WTAR = Wechsler Test for Adult Reading; WMS-III = Wechsler Memory Scale; HADS = Hospital Anxiety & Depression Rating Scale; LARS = Lille Apathy Rating Scale; SF-36 = Short Form-36; CRT = Choice reaction time; RT = Reaction time; s = seconds;

Domain	Neuropsychological Test	Healthy Controls Mean (+/- SD)	TBI Patients Mean (+/- SD)	W	p-uncorrected	p-FDR corrected
**Processing speed**	Trail Making Test A (s)	20.59 (5.78)	31.7 (17.61)	194	0.036	0.04
	Trail Making Test B (s)	50.13 (25.75)	67.23 (33.65)	188	0.027	0.03
	Stroop Colour Naming & Word Reading Composite Score (s)	26.63 (6.22)	31.23 (7.78)	188.5	0.028	0.8
	CRT Median RT (s)	0.43 (0.06)	0.53 (0.9)	115.5	<0.001	<0.01
**Executive Function**	Trail Making Test B-A (s)	32.5 (21.85)	34.57 (23.44)	288	0.82	0.82
	Stroop Inhibition (s)	50.94 (13.75)	61.37 (15.8)	185.6	0.024	0.03
	Stroop Inhibition-Switching (s)	58 (14.78)	73.2 (17.56)	126	<0.001	<0.01
	Stroop Inhibition-Switching vs. Baseline Contrast (s)	31.37 (15.03)	41.97 (13.09)	149.5	0.003	0.03
**Memory**	WMS-III Immediate Recall (Total I)	50.95 (6.97)	34.87 (11.55)	497.5	<0.001	<0.01
	WMS-III Delayed Recall (Total II)	32.26 (6.38)	20.6 (10.96)	471	<0.001	<0.01

	People Test Immediate Recall	28.85 (5.56)	23.4 (7.06)	451.5	<0.01	<0.01
	People Test Delayed Recall	10.2 (2.33)	8.3 (3.47)	394	0.05	0.06
**Intellectual ability**	WASI Matrix reasoning	29.15 (4.79)	27.67 (3.57)	379	0.11	0.1
	WTAR Scaled	116.5 (5.5)	108 (11.22)	449.5	<0.01	<0.01
**Behavioural reports**	LARS Self Total (apathy)	-33.53 (2.75)	-23.28 (9.52)	50.5	<0.001	<0.01
	HADS-D	3.33 (2.87)	7.07 (4.34)	105.5	<0.001	<0.01
	HADS-A	5.27 (3.43)	8.41 (4.79)	135	0.041	0.04
	SF-36 General Health	78.67 (16.2)	55.86 (23.07	342	0.002	<0.01
	SF-36 Physical Functioning	96.33 (4.81)	70.86 (24.28)	365	<0.001	<0.01
	SF-36 Emotional Wellbeing	78.13 (12.73)	61.93 (20.75)	322.5	0.009	0.01
	SF-36 Social Functioning	88.33 (16)	59.91 (20.15)	377.5	<0.001	<0.01
	SF-36 Energy/Fatigue	63.67 (16.53)	39.48 (21.06)	354.5	<0.001	<0.01

Supplementary Table 2: Clinical demographics

Study ID	Group	Age	Gender	Time since Injury (months)	Length of PTA (Days)	Cause of Injury	Extracranial Injury Requiring Hospital	Days in Hospital	Site of impact	Lowest GCS	Length of LOC	Initial CT findings	Initial MRI Findings	GOSE	UPDRS	Past Medical History	Medications at Visit
DREAM016	TBI	46	M	37	15	RTA	Yes	14	Frontal, Right	14	1-29 minutes	SAH: Left Sylvian Fissure	Microbleeds: Parafalcine, Left Frontal, Right Frontal	7	0	Gastro-oesophageal reflux disease	None
DREAM017	TBI	20	M	23	60	RTA	Yes	50	Occipital	13	1-29 minutes	SAH; Fracture: Left Occipital Bone; Contusion: Left Frontal, Right Frontal, Left Temporal, Right Temporal, Left Parietal, Right Parietal; SDH: Right Parieto-Temporal	Contusion: Right Temporal Pole, Right Frontal Pole; Microhaemorrhage: Left Parietal Lobe, Left Frontal Lobe, Right Frontal Lobe, Left Temporal Lobe, Left Occipital Lobe	5	0	None	None

Study ID	Group	Age	Gender	Time since Injury (months)	Length of PTA (Days)	Cause of Injury	Extracranial Injury Requiring Hospital	Days in Hospital	Site of impact	Lowest GCS	Length of LOC	Initial CT findings	Initial MRI Findings	GOSE	UPDRS	Past Medical History	Medications at Visit
DREAM021	TBI	52	M	43	5	RTA	Yes	42	Unknown	15	0 - 1 Minute	Normal	Microhaemorrhage: Parafalcine, Left Frontal, Right Frontal	6	0	None	None
DREAM022	TBI	45	M	32	14	Other Non Intentional	No	0	Frontal, Occipital	15	0 - 1 Minute	Not done	Normal	6	0	None	None
DREAM025	TBI	31	M	73	90	Violence	No	120	Unknown	6	Unknown	Fracture: Right orbital wall and floor, Right zygomatic arch	Contusion: Left Temporal Pole, Right Temporal Pole; Atrophy: Cerebellum; Superficial Siderosis: Cerebellum; Microhaemorrhage: Right Temporal Lobe, Left Frontal Lobe	5	22	None	None

Study ID	Group	Age	Gender	Time since Injury (months)	Length of PTA (Days)	Cause of Injury	Extracranial Injury Requiring Hospital	Days in Hospital	Site of impact	Lowest GCS	Length of LOC	Initial CT findings	Initial MRI Findings	GOSE	UPDRS	Past Medical History	Medications at Visit
DREAM026	TBI	49	M	249	120	Other Non Intentional	Yes	120	Frontal, Right	3	None	Unknown (in Bahamas)	Atrophy: Global, cerebellar, brain stem; Contusion: Right Frontal, Left Frontal, Right Temporal; Microhaemorrhage: Right Frontal, Left Frontal, Right Temporal	5	3	Rosacea	Sildenafil PRN, Loperamide PRN
DREAM028	TBI	24	M	8	14	RTA	Yes	15	Right	Unknown	Unknown	Normal	Microhaemorrhage: Parafalcine, Right Frontal, Left Frontal, Left Parietal, Left lentiform nucleus, Left Temporal, Right Temporal	6	0	None	None

Study ID	Group	Age	Gender	Time since Injury (months)	Length of PTA (Days)	Cause of Injury	Extracranial Injury Requiring Hospital	Days in Hospital	Site of impact	Lowest GCS	Length of LOC	Initial CT findings	Initial MRI Findings	GOSE	UPDRS	Past Medical History	Medications at Visit
DREAM033	TBI	52	M	17	21	Incident/ Fall	No	85	Occipital, Right	8	1-29 minutes	SDH: Large Left with midline shift; SAH: Bilateral; Contusion: Left Frontal, Right Occipital; Fracture: Occipital bone, Sphenoid bone	Contusion: Left Temporal Lobe, Left Frontal Lobe, Right Frontal Lobe, Left Cerebellar Hemishere; Microhaemorrhage: Left Temporal, Left Frontal, Right Temporal, Right Frontal; Superficial Siderosis: Left Frontal, Left Temporal	6	0	None	Levetirecetam 1250mg BD, Perindopril 2mg OD

Study ID	Group	Age	Gender	Time since Injury (months)	Length of PTA (Days)	Cause of Injury	Extracranial Injury Requiring Hospital	Days in Hospital	Site of impact	Lowest GCS	Length of LOC	Initial CT findings	Initial MRI Findings	GOSE	UPDRS	Past Medical History	Medications at Visit
DREAM037	TBI	54	M	11	15	Violence	No	20	Right	12	1-24 Hours	SDH: Right; SAH: Left Frontal, Right Frontal; Contusion: Left Frontal, Left Temporal, Right Temporal, Right Occipital	Contusion: Right Temporal, Right Parietal, Right Frontal, Left Frontal, Left Temporal; Superficial Siderosis: Left Frontal	5	0	None	None
DREAM038	TBI	26	M	17	56	Violence	No	49	Left, Occipital	Unknown	None	Contusion: Left Frontal, Left Temporal, Left Parietal; SAH: Left Parietal; SDH: Left	Contusion: Left Frontal, Left Parietal; Superficial Siderosis: Left Parietal, Left Frontal	5	0	None	Amitriptylline 10mg OD, Sodium Valproate 100mg OD

Study ID	Group	Age	Gender	Time since Injury (months)	Length of PTA (Days)	Cause of Injury	Extracranial Injury Requiring Hospital	Days in Hospital	Site of impact	Lowest GCS	Length of LOC	Initial CT findings	Initial MRI Findings	GOSE	UPDRS	Past Medical History	Medications at Visit
DREAM039	TBI	21	F	14	35	RTA	Yes	49	Unknown	14	Unknown	Normal	Microhaemorrhage: Left Frontal, Corpus Callosum, Right Frontal	6	0	None	Fluoxetine 20mg OD, Propranolol 10mg PRN
DREAM040	TBI	39	F	6	2	Incident/ Fall	No	3	Left, Frontal	15	0 - 1 Minute	SDH: Tentorium cerebelli	Normal	6	0	None	None
DREAM041	TBI	54	M	24	1	Incident/ Fall	No	5	Unknown	10	Unknown	Contusion: Left Temporal Lobe; SAH: Right Parietal, Left Temporal; Fracture: Left Squamous Temporal Bone	Contusion: Left Temporal Lobe; Microhaemorrhage: Right Temporal	8	0	Inflammatory Bowel Disease	Vitamin D

Study ID	Group	Age	Gender	Time since Injury (months)	Length of PTA (Days)	Cause of Injury	Extracranial Injury Requiring Hospital	Days in Hospital	Site of impact	Lowest GCS	Length of LOC	Initial CT findings	Initial MRI Findings	GOSE	UPDRS	Past Medical History	Medications at Visit
DREAM042	TBI	47	M	51	3	RTA	No	4	Right, Occipital	14	1-29 minutes	SAH: Bilateral; SDH: Right Frontal, Right Parietal, Left Frontal, Left Parietal; Fracture: Right Temporal Bone	Contusion: Right Parietal Lobe; Microhaemorrhage: Subcortical; Superficial Siderosis: Right Parietal Lobe, Left Frontal Lobe	6	0	None	Atorvastatin 10mg OD, Growth Hormone

Study ID	Group	Age	Gender	Time since Injury (months)	Length of PTA (Days)	Cause of Injury	Extracranial Injury Requiring Hospital	Days in Hospital	Site of impact	Lowest GCS	Length of LOC	Initial CT findings	Initial MRI Findings	GOSE	UPDRS	Past Medical History	Medications at Visit
DREAM044	TBI	22	M	21	540	RTA	Yes	90	Crown	3	> 7 Days	SAH: Right Frontal, Left Frontal; Contusion: Splenium, Left Temporal	Contusion: Left Frontal, Right Frontal, Splenium, Left Thalamus, Left Temporal; Atrophy: Global; Microhaemorrhage: Subcortical, Parafalcine, Left Temporal, Right Temporal, Splenium, Right Frontal, Left Cerebellar	4	33	None	None

Study ID	Group	Age	Gender	Time since Injury (months)	Length of PTA (Days)	Cause of Injury	Extracranial Injury Requiring Hospital	Days in Hospital	Site of impact	Lowest GCS	Length of LOC	Initial CT findings	Initial MRI Findings	GOSE	UPDRS	Past Medical History	Medications at Visit
DREAM046	TBI	48	M	366	60	RTA	Yes	60	Frontal	3	> 7 Days	Unknown	Contusion: Parafalcine, Left Frontal, Right Frontal; Microhaemorrhage: Left Temporal	4	0	Arthritis	Amitrptylline 20mg TDS, Esomeprazole 40mg OD, Solifenacin 5mg TDS, Botox injections, Gabapentin 600mg TDS
DREAM047	TBI	38	M	210	720	Violence	No	180	Unknown	3	> 7 Days	Unknown	Contusion: Left Occipital; Atrophy: Cerebellar, Pons, Left hemisphere	5	7	None	None
DREAM048	TBI	49	F	158	7	Violence	No	7	Left, Occipital	Unknown	1-29 minutes	Unknown	Normal	5	0	Hypothyroid	Levothyroxine 150 mcg OD, Omeprazole 20mg OD, Pyridoxine

Study ID	Group	Age	Gender	Time since Injury (months)	Length of PTA (Days)	Cause of Injury	Extracranial Injury Requiring Hospital	Days in Hospital	Site of impact	Lowest GCS	Length of LOC	Initial CT findings	Initial MRI Findings	GOSE	UPDRS	Past Medical History	Medications at Visit
DREAM052	TBI	36	F	219	120	RTA	No	160	Frontal	Unknown	Unknown	Unknown	Contusion: Right Temporal Lobe; Microhaemorrhage: Right Frontal Lobe, Posterior Limb Right Internal Capsule, Right Occipital Lobe; Atrophy: Cerebellum, Pons	4	43	None	None

Study ID	Group	Age	Gender	Time since Injury (months)	Length of PTA (Days)	Cause of Injury	Extracranial Injury Requiring Hospital	Days in Hospital	Site of impact	Lowest GCS	Length of LOC	Initial CT findings	Initial MRI Findings	GOSE	UPDRS	Past Medical History	Medications at Visit
DREAM053	TBI	43	M	6	42	RTA	Yes	66	Facial	3	1-29 minutes	SDH: Right; Contusion: Right Frontal Lobe	Superficial Siderosis: Right Frontal Lobe (vertex); Microhaemorrhage: Right Frontal lobe, Corpus Callosum (Genu and Splenium), Mid Brain.	5	0	None	None
DREAM054	TBI	31	M	22	21	Violence	No	4	Right, Occipital	Unknown	1-29 minutes	Contusion: Right Frontal, Right Temporal Lobe	Contusion: Right Frontal, Right Temporal Lobe	8	0	None	Lamotrigine 50mg BD, Growth Hormone 2 mg OD.

Study ID	Group	Age	Gender	Time since Injury (months)	Length of PTA (Days)	Cause of Injury	Extracranial Injury Requiring Hospital	Days in Hospital	Site of impact	Lowest GCS	Length of LOC	Initial CT findings	Initial MRI Findings	GOSE	UPDRS	Past Medical History	Medications at Visit
DREAM055	TBI	37	M	205	120	Incident/ Fall	Yes	150	Right	3	> 7 Days	Unknown	Contusion: Right Frontal Lobe, Left Frontal Lobe, Left Temporal Lobe; Atrophy: Corpus Callosum, Left Temporal Lobe, Right Cerebral Peduncle; Previous Right Frontal Craniectomy	5	0	None	Tegetrol CR 400mg BD

Study ID	Group	Age	Gender	Time since Injury (months)	Length of PTA (Days)	Cause of Injury	Extracranial Injury Requiring Hospital	Days in Hospital	Site of impact	Lowest GCS	Length of LOC	Initial CT findings	Initial MRI Findings	GOSE	UPDRS	Past Medical History	Medications at Visit
DREAM056	TBI	33	M	147	28	RTA	No	30	Left, Occipital	5	Unknown	Fracture: Left Squamous Temporal Bone; Contusion: Right Frontal Lobe, Left Frontal Lobe, Left Temporal Lobe; SAH: Left Frontal, Left Temporal Lobes	Contusion: Right Frontal Lobe; Microhaemorrhage: Parafalcine, Right Frontal, Left Frontal; High Signal: Periventricular and Pontine, inkeeping with demyelination	6	11	None	Baclofen 20mg OD, Citalopram 10mg OD

Study ID	Group	Age	Gender	Time since Injury (months)	Length of PTA (Days)	Cause of Injury	Extracranial Injury Requiring Hospital	Days in Hospital	Site of impact	Lowest GCS	Length of LOC	Initial CT findings	Initial MRI Findings	GOSE	UPDRS	Past Medical History	Medications at Visit
DREAM057	TBI	34	M	9	7	Incident/ Fall	No	3	Right, Occipital	10	1-24 Hours	Normal	Contusion: Left Caudate Head; Microhaemorrhage: Left Temporal Pole, Left Occipital Lobe, Parafalcine (Left and Right Frontal Lobes)	6	0	None	None

Study ID	Group	Age	Gender	Time since Injury (months)	Length of PTA (Days)	Cause of Injury	Extracranial Injury Requiring Hospital	Days in Hospital	Site of impact	Lowest GCS	Length of LOC	Initial CT findings	Initial MRI Findings	GOSE	UPDRS	Past Medical History	Medications at Visit
DREAM060	TBI	32	M	73	28	RTA	Yes	28	Unknown	3	> 7 Days	EDH: Right Temporal; Fracture: Right Temporal Bone, Right Greater Wing of Sphenoid, Left Frontal Bone, Left Greater Wing of Sphenoid, Left Maxillary Wall, Left Zygomatic Arch; SAH: Right Vertex	EDH: Left Frontal, Right Temporal, Left Occipital; SAH: Right Vertex; Contusion: Left Frontal Lobe; High Signal: Splenium; Microhaemorrhage: Left Frontal Lobe, Right Frontal Lobe	6	0	Asthma	Symbicort

Study ID	Group	Age	Gender	Time since Injury (months)	Length of PTA (Days)	Cause of Injury	Extracranial Injury Requiring Hospital	Days in Hospital	Site of impact	Lowest GCS	Length of LOC	Initial CT findings	Initial MRI Findings	GOSE	UPDRS	Past Medical History	Medications at Visit
DREAM061	TBI	52	M	23	4	Violence	No	3	Occipital	14	Unknown	Fracture: Occipital bone	Contusion Right Frontal Lobe, Left Frontal Lobe, Left Temporal Lobe, Right Temporal Lobe	6	0	Gastro-oesophageal reflux disease	Lanzoprazole 15 mg OD
DREAM063	TBI	38	M	9	14	Violence	No	14	Occipital, Crown	3	1-24 Hours	Contusion: Left Frontal Lobe, Right Frontal Lobe, Right Temporal Lobe; SDH: Parafalcine; SAH: Parafalcine; Fracture: Right Occipital Bone	Contusion: Right Frontal Lobe (superior frontal gyrus and frontal pole), Right Temporal Lobe (temporal pole), Left Frontal Lobe to a lesser degree; Superficial Siderosis: Vertex, right worse than left; Microhaemorrhages: Vertex, right worse than left	6	0	None	None

Study ID	Group	Age	Gender	Time since Injury (months)	Length of PTA (Days)	Cause of Injury	Extracranial Injury Requiring Hospital	Days in Hospital	Site of impact	Lowest GCS	Length of LOC	Initial CT findings	Initial MRI Findings	GOSE	UPDRS	Past Medical History	Medications at Visit
DREAM064	TBI	31	M	112	120	RTA	No	71	Occipital	6	> 7 Days	Contusion: Left Frontal Lobe, Right Frontal Lobe, Corpus Callosum, Right Thalamus	Contusion: Parafalcine (Right and Left Frontal Lobes, worse on Right), Right Thalamus, Right and Left Parietal Lobes; Microhaemorrhage: Left Frontal Lobe, Right Frontal Lobe, Right Temporal Pole, Left Hippocampus; Atrophy: Cerebellum and globally	5	11	None	None

Study ID	Group	Age	Gender	Time since Injury (months)	Length of PTA (Days)	Cause of Injury	Extracranial Injury Requiring Hospital	Days in Hospital	Site of impact	Lowest GCS	Length of LOC	Initial CT findings	Initial MRI Findings	GOSE	UPDRS	Past Medical History	Medications at Visit
DREAM065	TBI	39	M	33	8	RTA	Yes	14	Frontal, Right	13	1-29 minutes	Contusion: Left Frontal Lobe	Contusion: Left Orbito-Frontal Lobe, Right Orbito-Frontal Lobe (worse on the left).	5	0	None	None
DREAM067	TBI	24	F	16	120	RTA	No	30	Occipital, Left	3	Unknown	Contusion: Right Thalamus, Left Frontal Lobe, Right Frontal Lobe, Left Occipital Lobe, Right Occipital Lobe; SDH: Vertex	Contusion: Left Frontal Lobe, Right Frontal Lobe, Left Temporal Lobe, Corpus Callosum; Microhaemorrhage: Right Parietal Lobe, Left Temporal Lobe, Corpus Callosum; Atrophy: Splenium and Genu of Corpus Callosum	6	1	None	Citalopram 40mg OD

Study ID	Group	Age	Gender	Time since Injury (months)	Length of PTA (Days)	Cause of Injury	Extracranial Injury Requiring Hospital	Days in Hospital	Site of impact	Lowest GCS	Length of LOC	Initial CT findings	Initial MRI Findings	GOSE	UPDRS	Past Medical History	Medications at Visit
DREAM069	TBI	35	M	7	3	Incident/ Fall	No	5	Left	Unknown	1-29 minutes	Contusion: Right Frontal Lobe; EDH: Left; Fracture: Left Parietal Bone	Contusion: Right Frontal Lobe, Left Temporal Lobe, Right Temporal Lobe	6	0	Asthma	Salbutamol inhalers
MEMCT04	Control	30	M														
MEMCT05	Control	27	M														
DREAM024	Control	43	M														

Study ID	Group	Age	Gender	Time since Injury (months)	Length of PTA (Days)	Cause of Injury	Extracranial Injury Requiring Hospital	Days in Hospital	Site of impact	Lowest GCS	Length of LOC	Initial CT findings	Initial MRI Findings	GOSE	UPDRS	Past Medical History	Medications at Visit
DREAM027	Control	49	M														
DREAM029	Control	44	M														
DREAM031	Control	45	M														
DREAM034	Control	29	M														

Study ID	Group	Age	Gender	Time since Injury (months)	Length of PTA (Days)	Cause of Injury	Extracranial Injury Requiring Hospital	Days in Hospital	Site of impact	Lowest GCS	Length of LOC	Initial CT findings	Initial MRI Findings	GOSE	UPDRS	Past Medical History	Medications at Visit
DREAM035	Control	34	M														
DREAM043	Control	46	M														
DREAM049	Control	47	F														
DREAM050	Control	33	M														

Study ID	Group	Age	Gender	Time since Injury (months)	Length of PTA (Days)	Cause of Injury	Extracranial Injury Requiring Hospital	Days in Hospital	Site of impact	Lowest GCS	Length of LOC	Initial CT findings	Initial MRI Findings	GOSE	UPDRS	Past Medical History	Medications at Visit
DREAM051	Control	25	M														
DREAM058	Control	35	M														
DREAM059	Control	24	M														
DREAM062	Control	35	M														

Study ID	Group	Age	Gender	Time since Injury (months)	Length of PTA (Days)	Cause of Injury	Extracranial Injury Requiring Hospital	Days in Hospital	Site of impact	Lowest GCS	Length of LOC	Initial CT findings	Initial MRI Findings	GOSE	UPDRS	Past Medical History	Medications at Visit
DREAM068	Control	57	M														
MEMCT01	Control	28	F														
MEMCT02	Control	35	M														
TauTBI_106	Control	29	F														

Study ID	Group	Age	Gender	Time since Injury (months)	Length of PTA (Days)	Cause of Injury	Extracranial Injury Requiring Hospital	Days in Hospital	Site of impact	Lowest GCS	Length of LOC	Initial CT findings	Initial MRI Findings	GOSE	UPDRS	Past Medical History	Medications at Visit
TauTBI202	Control	62	M														
